# Interferon alpha-induced uPAR expression on monocytes as a potential source of increased soluble uPAR in patients with SLE at high risk of developing organ damage

**DOI:** 10.1007/s10238-025-01897-4

**Published:** 2025-10-21

**Authors:** Lina Wirestam, Jesper Karlsson, Astrid Welin, Jhonatan Antonio Álvarez-Gómez, Jonas Wetterö, Christopher Sjöwall, Helena Enocsson

**Affiliations:** Division of Inflammation and Infection, Department of Biomedical and Clinical Sciences, Linköping University, Linköping, Sweden

**Keywords:** Urokinase plasminogen activator receptor, suPAR, Systemic lupus erythematosus, Damage accrual, Inflammation

## Abstract

The soluble urokinase plasminogen activator receptor (suPAR) has been shown to adequately predict the risk of organ damage development in patients with systemic lupus erythematosus (SLE). However, the cellular source and mechanism of increased circulating levels remain unknown. The cell-bound uPAR is expressed on various cell types and can be shed from the plasma membrane surface by proteases, resulting in the soluble form, suPAR. Herein, leukocyte uPAR expression and plasma suPAR levels were explored in unstimulated and cytokine-treated (tumor necrosis factor; TNF or interferon-α; IFN-α) blood from patients with SLE (*n* = 37) and healthy blood donors (HBD; *n* = 27). Unstimulated or cytokine-treated whole blood was thereafter used for analysis of cellular uPAR expression by flow cytometry, and plasma suPAR levels by enzyme-linked immunosorbent assay (ELISA). Monocytes and neutrophils had the most prominent uPAR expression and showed increased expression after TNF addition to the blood (*p* < 0.001). Addition of IFN-α resulted in increased uPAR expression only in patients (*p* < 0.05). No differences in uPAR expression were found depending on organ damage or other clinical variables. Nevertheless, suPAR levels were higher in patients with organ damage (*p* < 0.01). Unstimulated suPAR levels were correlated with monocyte uPAR expression in patient samples (rho = 0.4). As circulating suPAR levels can predict organ damage development in SLE, it is important to unravel its potential disease mediating effects as well as cellular sources. Results of this study suggest IFN-α as a regulator of uPAR expression in SLE and monocyte uPAR as an important source of plasma suPAR.

**Supplementary Information:**

The online version contains supplementary material available at 10.1007/s10238-025-01897-4.

## Introduction

Systemic lupus erythematosus (SLE) is a systemic inflammatory autoimmune disease characterized by inadequate clearance of apoptotic debris, loss of immune tolerance and production of antinuclear autoantibodies (ANA) [1]. Immune complexes with nucleic acids as well as neutrophil extracellular trap (NET) formation can activate the type I interferon (IFN) system, which constitutes a hallmark of the disease. The inflammatory milieu has the potential to attenuate immune dysregulation and cell death, creating a vicious circle of immune activation [1].

Over time, consequences of the disease, drug-related side effects and age will give rise to irreversible organ damage that strongly associates with increased mortality [2–4] and reduced quality of life [5–7]. Organ damage in SLE has been defined by the Systemic Lupus International Collaborating Clinics/American College of Rheumatology damage index (SDI) and includes descriptors in 12 organ domains [2, 8]. The SDI instrument measures any organ damage that has occurred since the onset of SLE, and the damage must have been present for at least 6 months.

The use of corticosteroids is strongly associated with increased risks of specific organ damage types, including osteoporosis-related fractures, cataract and diabetes. In contrast, disease activity is the primary contributor to other forms of organ damage, such as cognitive dysfunction and chronic kidney disease [9]. Demographic factors such as higher age, non-Caucasian ethnicity and male sex are associated with the increased risk of damage accrual [10–12].

Despite the clinical importance of preventing the development of organ damage, there are currently no biomarkers in clinical practice that provides prognostic information regarding the risk of damage accrual in SLE. The previous research from our group has identified a correlation between elevated blood levels of soluble urokinase plasminogen activator receptor (suPAR) and both the presence of organ damage [13], and association to the progressive accrual of organ damage [14].

SuPAR originates from cell-bound uPAR (also named CD87), which is attached to the cell membrane via a glycosyl phosphatidylinositol (GPI) anchor and encoded by the PLAUR gene. Cell surface uPAR expression has been reported in tissues undergoing remodeling such as embryo implantation, wound healing and cancer metastasis where it contributes to cell adhesion and migration [15]. Immune cells, including monocytes and neutrophils, also express uPAR [16, 17]. In the context of SLE, it is interesting to note that uPAR seems to be involved in the regulation of apoptotic cell phagocytosis, i.e., efferocytosis [18, 19]. During immune activation, uPAR can undergo cleavage by proteases such as neutrophil elastase, urokinase plasminogen activator (uPA), plasmin or cathepsin G. This cleavage results in the release of suPAR, which has been detected in saliva, plasma, serum, urine and cerebral spinal fluid [20, 21]. We, and others, have found associations between suPAR and disease outcomes in different inflammatory diseases [13, 14, 22–24], but the biological mechanisms underlying these findings remain largely unknown. Shedding of uPAR from neutrophils was suggested as a source of suPAR in critical illness [24], but to our knowledge, the source of suPAR in other diseases is not studied. Apart from serving as a biomarker, suPAR is also suggested as a causative agent, affecting podocytes, in focal segmental glomerulosclerosis [25], and lupus nephritis [26].

This study aimed to investigate the relationship between plasma membrane uPAR expression and blood plasma suPAR levels in patients with SLE and healthy controls, as well as to examine the effects of immune activation with tumor necrosis factor (TNF) and IFN-α (a key cytokine in SLE) on uPAR expression and suPAR levels. uPAR expression and suPAR concentrations were also assessed in relation to clinical phenotypes of the patients.

## Methods

### Patients and healthy blood donors

We included 37 consecutive patients meeting the 1982 American College of Rheumatology (ACR) and/or the 2012 SLICC classification criteria for SLE [27] at the Linköping University Hospital, who participated in a prospective quality and research register [28]. Organ damage was assessed by the SDI [8] and disease activity by the Systemic Lupus Erythematosus Disease Activity Index 2000 (SLEDAI-2 K) [29]. For comparison, healthy blood donors (HBD), matching the patients regarding age and sex, served as controls. Baseline characteristics of the patients and HBD are detailed in Table 1.

**Table 1.** Clinicodemographic data of patients and controls.

	Median (range) or number (%)		
	SLE (*n*=37)	HBD (*n*=27)	P-value
Age (years)	44 (23–86)	50 (22–64)	0.549
Sex (female)	31 (84%)	20 (74%)	0.502
Disease duration (years)	14 (0–32)	NA	NA
Organ damage (SDI score)	1 (0–5)	NA	NA
Organ damage (SDI≥1)	19 (51%)	NA	NA
Disease activity (SLEDAI-2K)	2 (0–12)	NA	NA
Fulfillment of ACR criteria		NA	NA
1. Malar rash	15 (41%)		
2. Discoid rash	1 (2.7%)		
3. Photosensitivity	19 (51%)		
4. Oral ulcers	6 (16%)		
5. Arthritis	31 (84%)		
6. Serositis	15 (41%)		
7. Renal disorder	11 (30%)		
8. Neurological disorder	3 (8.1%)		
9. Hematologic disorder	26 (70%)		
10. Immunologic disorder	24 (65%)		
11. Antinuclear antibody	37 (100%)		
Ongoing therapy		NA	
Prednisolone dose (mg/day)	2.5 (0–7.5)		
Prednisolone (≥5 mg/day)	15 (41%)		
Hydroxychloroquine	33 (89%)		
Mycophenolate mofetil	9 (24%)		
B-cell targeted biologic (Belimumab or Rituximab)	5 (14%)		
Methotrexate	4 (11%)		
Azathioprine	4 (11%)		
Anifrolumab	1 (2.7%)		

### Blood sampling and treatment with TNF and IFN-α

Peripheral whole blood samples, with EDTA as anticoagulant, were collected from patients and HBD. One portion of the blood was sent for analysis of blood cell concentrations at the Clinical chemistry unit at the Linköping University Hospital. The other portion of blood was treated with TNF (10 ng/ml) from R&D Systems, Biotechne, Minneapolis, MN, USA) or IFN-α2b (100 or 1000 U/ml) from PBL assay sciences, Piscataway, NJ, USA, or was left untreated. The samples were thereafter incubated in a 37 °C water bath for 20 min [30]. Depending on the obtained blood volume and technical issues, not all samples were treated with both TNF and IFN-α2b (hereafter denoted IFN-α). Blood from the patient with ongoing anifrolumab treatment (targeting the IFN-α/β receptor; IFNAR) was not used in IFN-α treatment experiments. All cytokine-treated samples had an untreated counterpart for pairwise comparison.

One portion of the cytokine-treated whole blood was analyzed by flow cytometry, and cells were labeled as described below.

The other portion of the cytokine-treated whole blood was centrifuged at 1500* g* for 10 min to generate plasma. The plasma was thereafter stored at − 80 °C until suPAR analysis by ELISA as described below.

### uPAR expression

An initial pilot study examined uPAR expression on multiple leukocytes (monocytes, neutrophils, T, B and NK-cells) from patients and HBD. Results from this pilot experiments revealed the highest expression of uPAR on monocytes and neutrophils (Supplementary Fig. 1), and these populations were subsequently chosen for continuous analyses of uPAR expression. Neutrophils are also by far the most abundant leukocytes in blood, thus contributing with the highest potential of uPAR/suPAR to the circulation.

For uPAR cell surface expression analysis, treated or untreated whole blood samples (as described above) were incubated for 30 min at 4 °C with flow cytometry antibodies. Anti-CD87/uPAR (1:20, clone VIM5, BV421, RRID AB_2741287) and anti-CD14 (1:5, clone M5E2, PE, RRID AB_395799) were from BD Bioscience, Franklin Lakes, New Jersey, US. Anti-CD15 (1:40, clone HI98, Alexa488, RRID AB_493257) was from Nordic Biosite, Stockholm, Sweden. Anti-CD62L (1:20, clone DREG56, PE-Cy7, RRID AB_1257142) and anti-CD11b (1:20, clone ICRF44, APC, RRID AB_2016659) were from ThermoFisher Scientific, Waltham, MA, USA). Erythrocytes were lysed by incubation with BD PharmLyse (BD Biosciences) for 15 min at room temperature, and the cells were thereafter washed and resuspended in PBS (0.1% FBS) prior to flow cytometry analysis (Gallios, Beckman Coulter Life Sciences, Indianapolis, Indiana, US). The flow cytometry data analysis was performed with Kaluza Analysis 2.2 (Beckman Coulter Life Sciences).

When uPAR expression on different cell types was related to the levels of plasma suPAR, the cell concentration of the blood (data from the clinical chemistry unit) was multiplied by the median fluorescence intensity (MFI) of uPAR of that particular cell type. This calculation was performed to reach a fair estimate of the total cell surface-bound uPAR pool for the respective cell type in the blood and is referred to as ‘total uPAR expression’ in the manuscript.

### Gating strategy

Gating strategy and flow cytometry antibodies in the pilot study are found in Supplementary Figs. 2 and 3. The antibody used for CD16 detection in the pilot study (3G8 clone, Alexa700 conjugated, IgG1; BioLegend, San Diego, CA, US) caused a substantial reduction of recorded events at flow cytometry analysis (data not shown) and was therefore not used for neutrophil gating in continuous experiments.

**Fig. 1:**
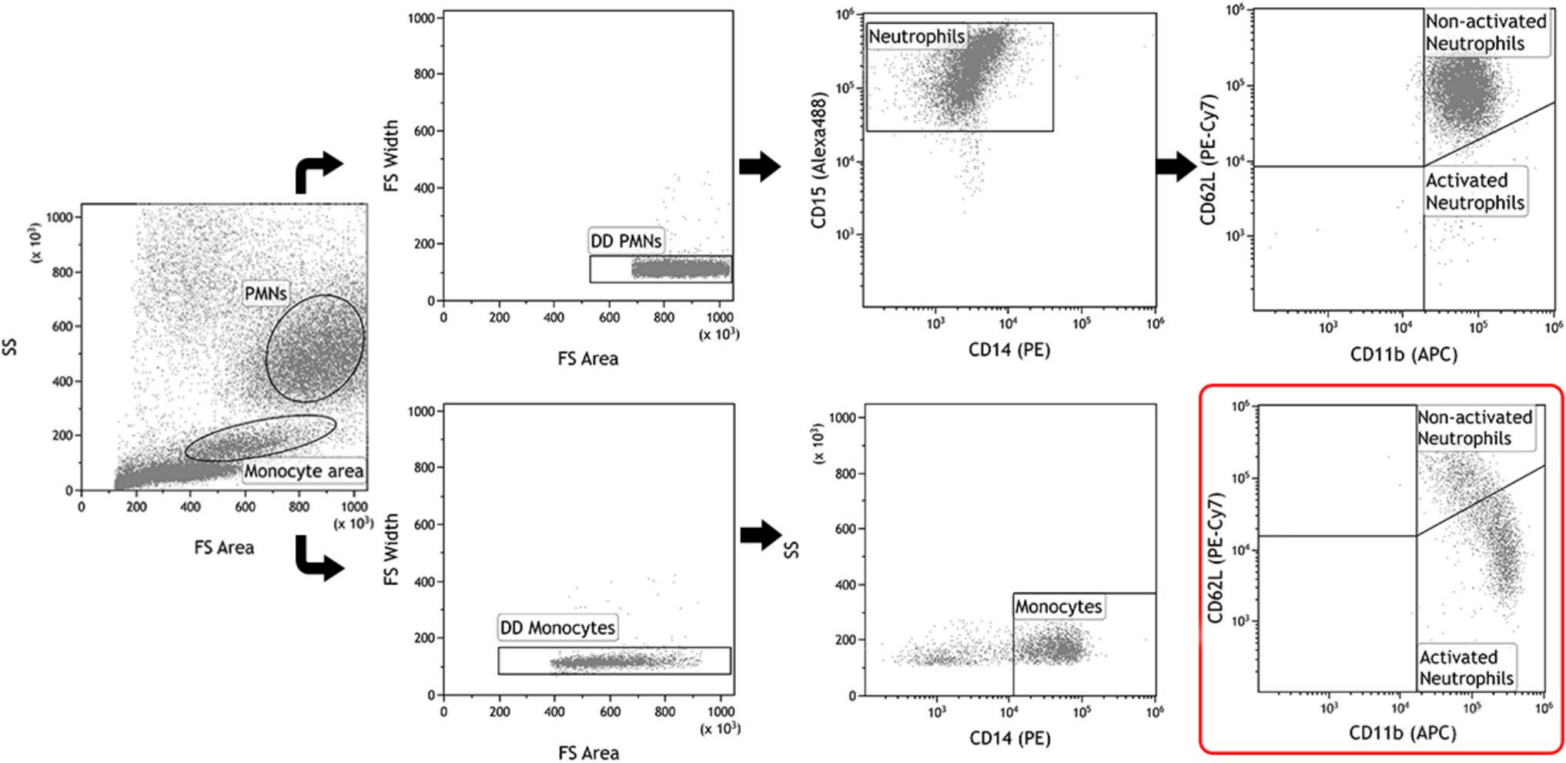
Flow cytometry gating strategy for neutrophils, monocytes and activated neutrophils. Polymorphonuclear leukocytes (PMNs) and monocytes were first located from total events using forward scatter (FS) area and side scatter (SS). Both PMNs and monocytes were gated through a double discrimination (DD) gate before defining neutrophils as CD15^+^CD14^−^ and monocytes as CD14^+^. A representative dot plot of neutrophils activated by TNF-stimulation is inserted in the red square

Flow cytometry gating strategy for neutrophils, monocytes and activated neutrophils in the present study is visualized in Fig. 1. Neutrophils were defined as CD15 ^+^ CD14^−^. Monocytes were defined as CD14 ^+^. In a separate gate, neutrophils were further identified as non-activated (CD11b^+^CD62L^high^) and activated (CD11b^high^CD62L^dim^). More specifically, activated neutrophils were defined by decreased CD62L expression and increased CD11b expression in comparison with its untreated counterpart. Expression of uPAR/CD87 for each cell population was obtained by subtracting the MFI of the control sample (fluorescence minus one; FMO) from the MFI of the uPAR-stained sample.

### suPAR plasma levels

SuPAR was analyzed by the clinically validated suPARnostic® ELISA (ViroGates, Birkerød, Denmark). The assay was run according to the manufacturer’s instructions, briefly described previously [31]. Control samples provided in the kit were within the acceptable range at all assays.

### Statistics

According to the Shapiro–Wilk test for normality, uPAR expression on monocytes was normally distributed, but uPAR expression on neutrophils as well as suPAR levels was not normally distributed. Therefore, nonparametric statistical tests were used consistently for the analysis of the data. Correlations were calculated using Spearman correlation tests. For comparisons between two groups, the Mann–Whitney *U* test was used for continuous data, whereas the Fisher’s exact test was used for categorical data. Kruskal–Wallis was applied for comparisons of three groups. Pairwise comparisons were made using Wilcoxon signed-rank test. Linear regression analysis was used for evaluation of the impact of variables with correlation to uPAR expression or suPAR levels. In these analyses, uPAR and suPAR were log10-transformed to obtain a normal distribution. A *P*-value of *p* < 0.05 was considered statistically significant. SPSS Statistics 29 (IBM Corp., Armonk, NY, USA) was used for statistical analyses, and GraphPad Prism Version 10.4 (GraphPad Software, Boston, MA, USA) was applied for graph generation.

## Results

### Unstimulated uPAR expression in SLE and HBD

**Fig. 2:**
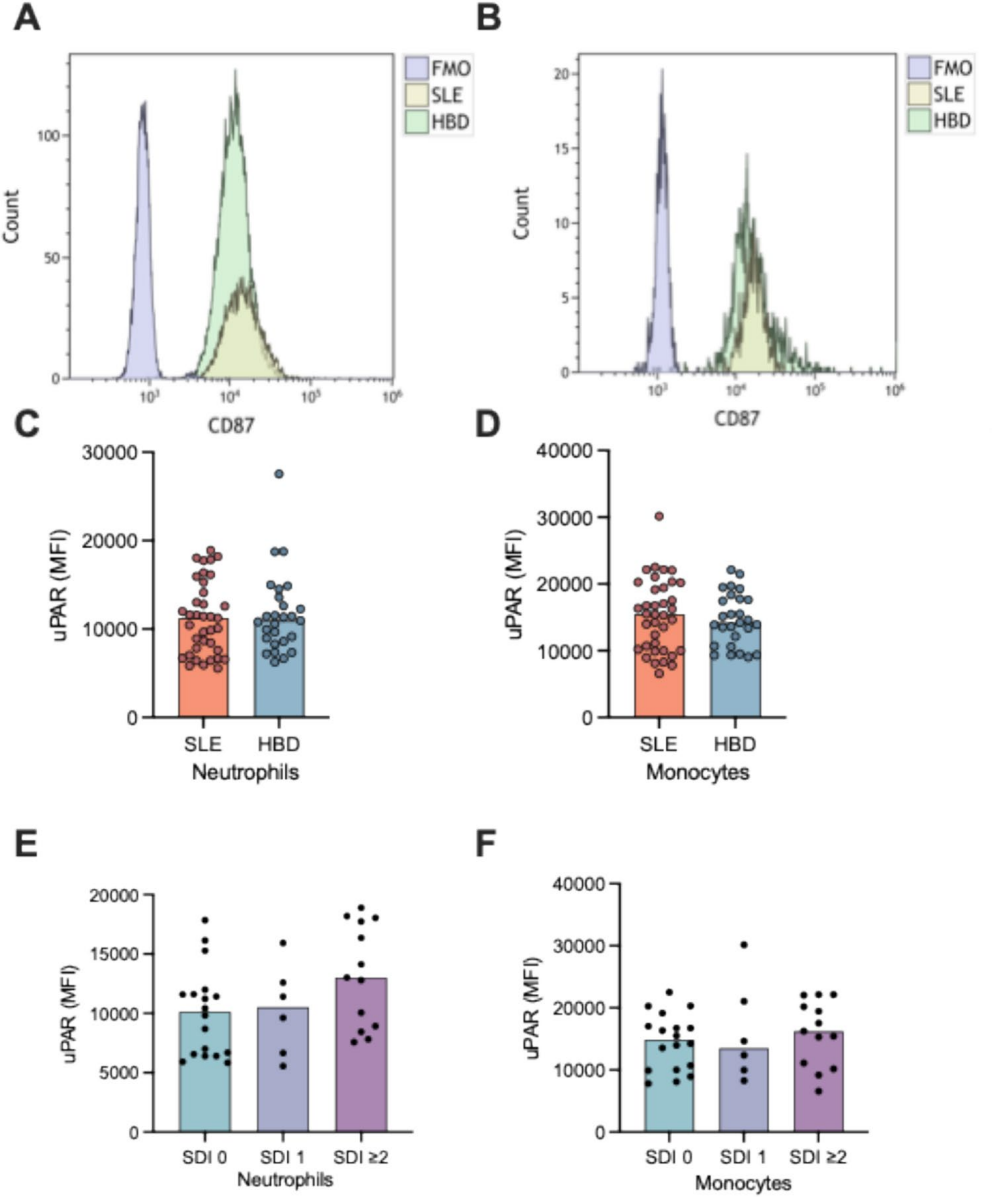
Unstimulated uPAR (CD87) expression on neutrophils and monocytes from systemic lupus erythematosus (SLE) patients and healthy blood donors (HBD). Representative histogram of the unstimulated uPAR expression on neutrophils (**a**) and monocytes (**b**). uPAR expression comparing patients and HBD on (**c**) neutrophils and (**d**) monocytes. uPAR expression from patients with different SLICC/ACR organ damage index (SDI) scores on (**e**) neutrophils and (**f**) monocytes. The bars display median values, and the dots represent each individual. FMO: fluorescence minus one.

Unstimulated cellular uPAR expression in subjects with SLE and HBD revealed the expression of uPAR on all monocytes (Fig. 2a) and neutrophils (Fig. 2b). However, the mean plasma membrane expression of uPAR did not differ between patients and HBD neither on monocytes, nor on neutrophils (Fig. 2c and d).

**Fig. 3:**
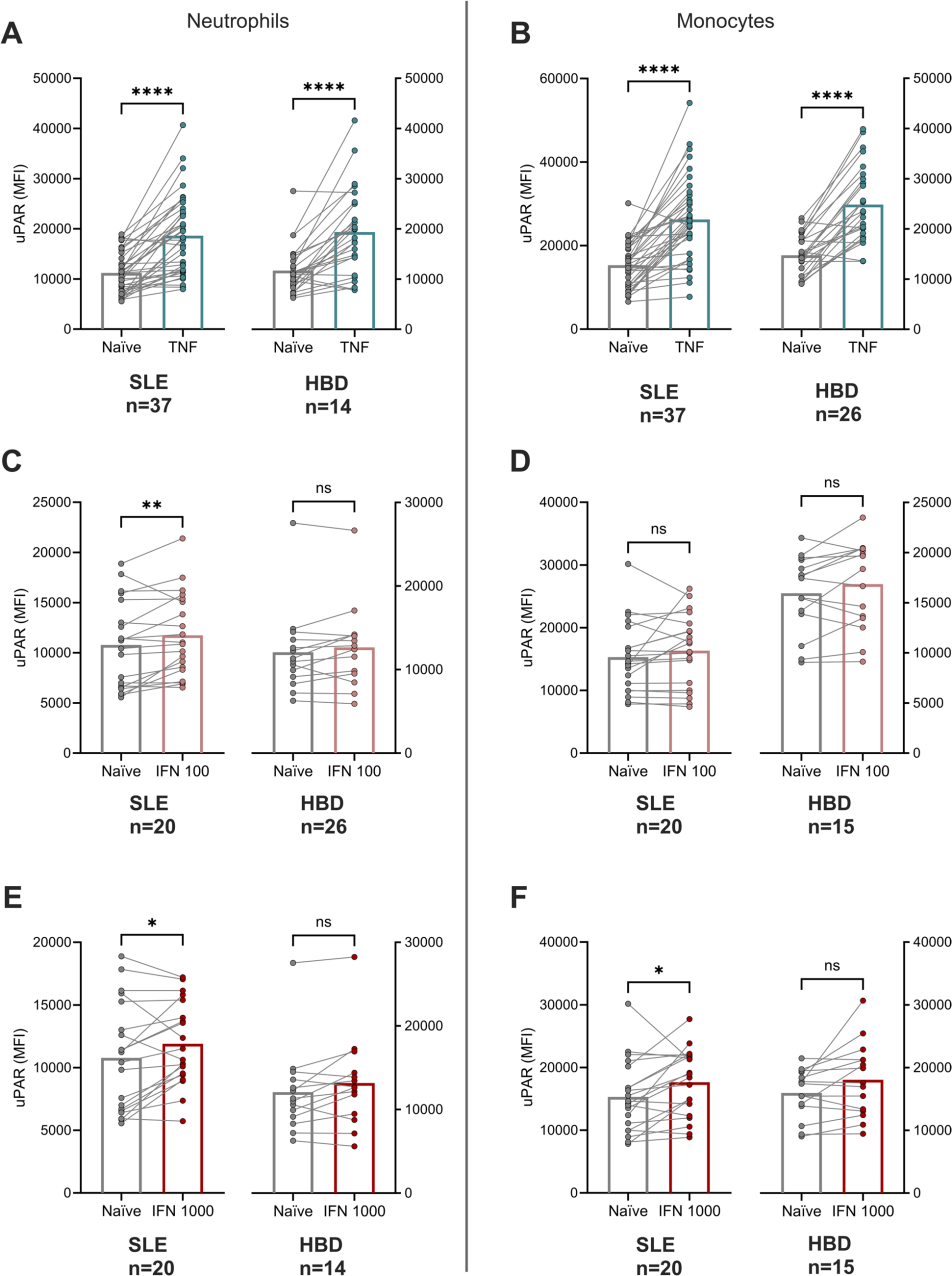
Effects of cytokine stimulation on neutrophil uPAR expression (**a, c, e**) and monocyte uPAR expression (**b, d, f**) in patients and healthy blood donors (HBD). TNF (A and B) and IFN-α in a low concentration (**c** and **d**) and high concentration (**e** and **f**) were used to treat the whole blood. Bars indicate median value, whereas dots represent each individual. Significances from paired tests are shown. *p < 0.05, **p < 0.01, ****p < 0.0001

To reveal patient-related factors that may associate with uPAR expression, the patients were divided based on presence of organ damage (none *vs.* any), renal disorder (none *vs.* history of renal disorder), as well as prednisolone dosages equivalents (< 5 mg vs. ≥ 5 mg). None of these comparisons revealed statistically significant differences in uPAR expression, neither on neutrophils, nor in monocytes (not shown). Results for uPAR expression with regard to the organ damage score (Figs. 2e and 3f) trended toward increased mean expression of uPAR on neutrophils dependent on organ damage score, but without reaching statistical significance (Fig. 2e). Furthermore, there was no significant difference in monocyte and neutrophil uPAR expression based on sex among patients or HBD. No association between monocyte and neutrophil uPAR expression and age among HBD was observed. However, among patients, a significant correlation was found between patient age and neutrophil (rho = 0.345, *p* = 0.036) and monocyte (rho = 0.358, *p* = 0.03) uPAR plasma membrane expression. In addition, significant correlations were found between disease duration and uPAR expression on neutrophils (rho = 0.535, *p* < 0.001) and on monocytes (rho = 0.525, *p* < 0.001). As age and disease duration are correlated, the impact of these two variables was investigated in a linear regression analysis (stepwise approach) with 10log uPAR as the dependent variable. In these analyses, age was removed from the model, leaving only disease duration as the variable with significant impact on monocyte (*p* = 0.003, Standardized beta = 0.42) and neutrophil (*p* = 0.003, Standardized beta = 0.53) uPAR expression. Regarding associations between uPAR and clinical variables of relevance for inflammation, we found no significant correlations with disease activity (SLEDAI-2 K scores), complement proteins (C3 or C4 concentrations), creatinine, erythrocyte sedimentation rate or C-reactive protein levels among patients.

### Cytokine-treated expression of uPAR in SLE and HBD

TNF treatment of whole blood revealed a significant increase of uPAR expression on both neutrophils and monocytes. This was observed both in the patient group and among HBD (Fig. 3a and b). Treatment with IFN-α was performed in two different concentrations where the lowest concentration (100 U/mL) resulted in a significant increase in uPAR expression on neutrophils in the patient group (Fig. 3c). Treatment with the higher concentration of IFN-α (1000 U/mL) significantly increased the uPAR expression on both neutrophils and monocytes in the patient group (Fig. 3e and f). Cells from HBD did not respond with uPAR upregulation upon IFN-α treatment.

### uPAR expression on activated and non-activated neutrophils

**Fig. 4:**
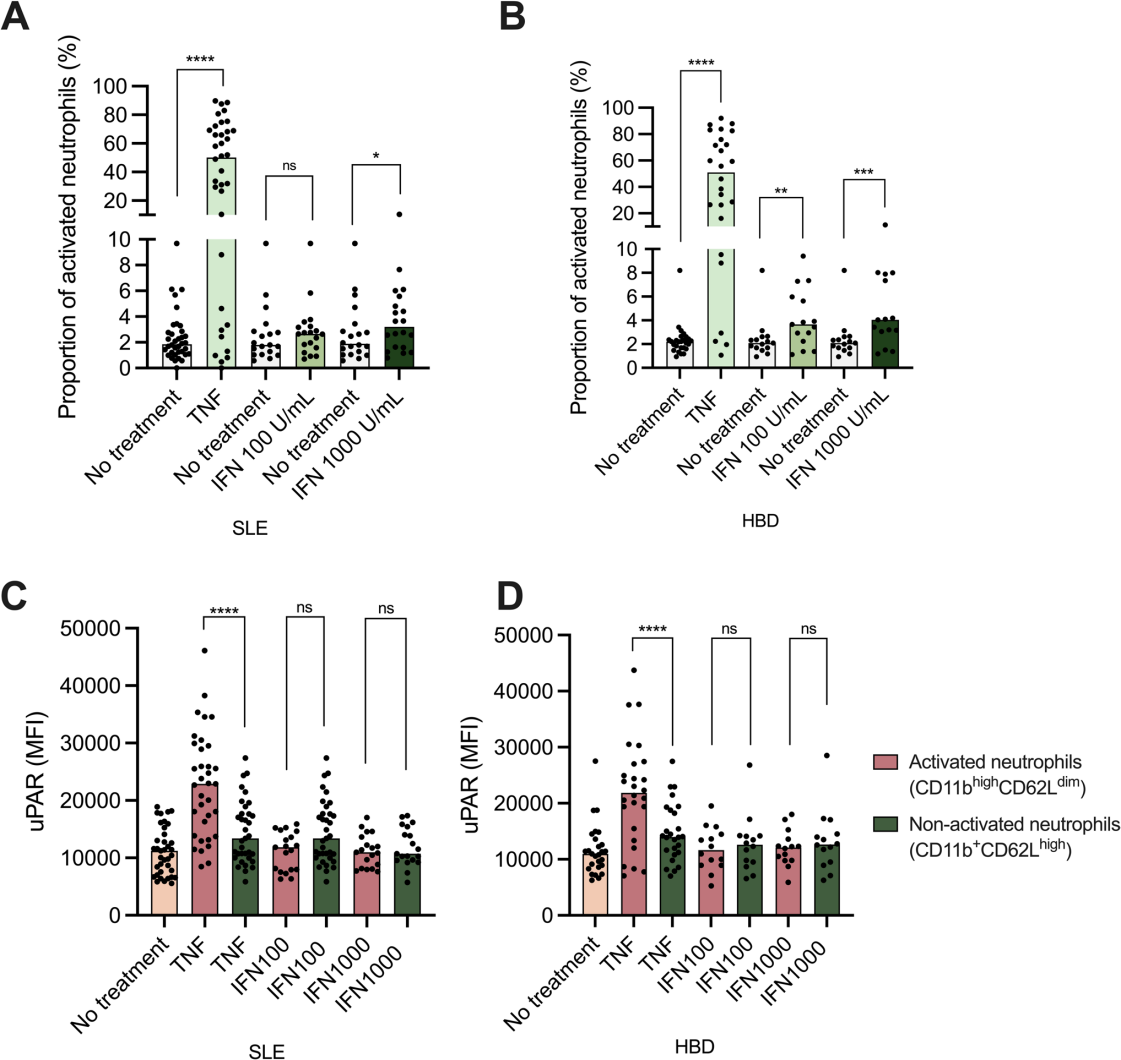
Proportion of activated neutrophils (CD11b^high^CD62L^dim^) (**a** and **b**) and the expression of uPAR on activated versus non-activated (CD11b^+^CD62L.^high^) (**c** and **d**) after treatment with TNF or IFN-α in two different concentrations (100 and 1000 U/mL). TNF: tumor necrosis factor, IFN: Interferon, SLE: systemic lupus erythematosus, HBD: healthy blood donors. The graphs display median values as well as individual measurements. Significances from paired tests are shown. *p < 0.05, **p < 0.01, **p < 0.001, ****p < 0.0001

The proportion of activated neutrophils in cytokine-treated samples and the expression of uPAR on non-activated (CD11b^+^CD62L^high^) *vs.* activated (CD11b^high^CD62L^dim^) neutrophils were investigated next. TNF-stimulation dramatically increased the proportion of activated neutrophils in blood from patients as well as HBD (Fig. 4a and b). IFN-α treatment had a more moderate effect on neutrophil activation, reaching statistical significance for both concentrations of IFN-α in HBD and for the highest concentration of IFN-α in SLE (Fig. 4a and b).

In TNF-treated blood, the uPAR expression was significantly increased in activated neutrophils in comparison with non-activated neutrophils (Fig. 4c and d). There was, however, no significant difference in uPAR expression between activated and non-activated neutrophils from blood that had been treated with IFN-α at any concentration (Fig. 4c and d). This was true for patients with SLE, as well as for HBD. Consequently, the increased uPAR expression on IFN-α-treated neutrophils, which we found in SLE samples (Fig. 3), was not only a result of neutrophil activation.

### Plasma suPAR in patients and HBD

**Fig. 5:**
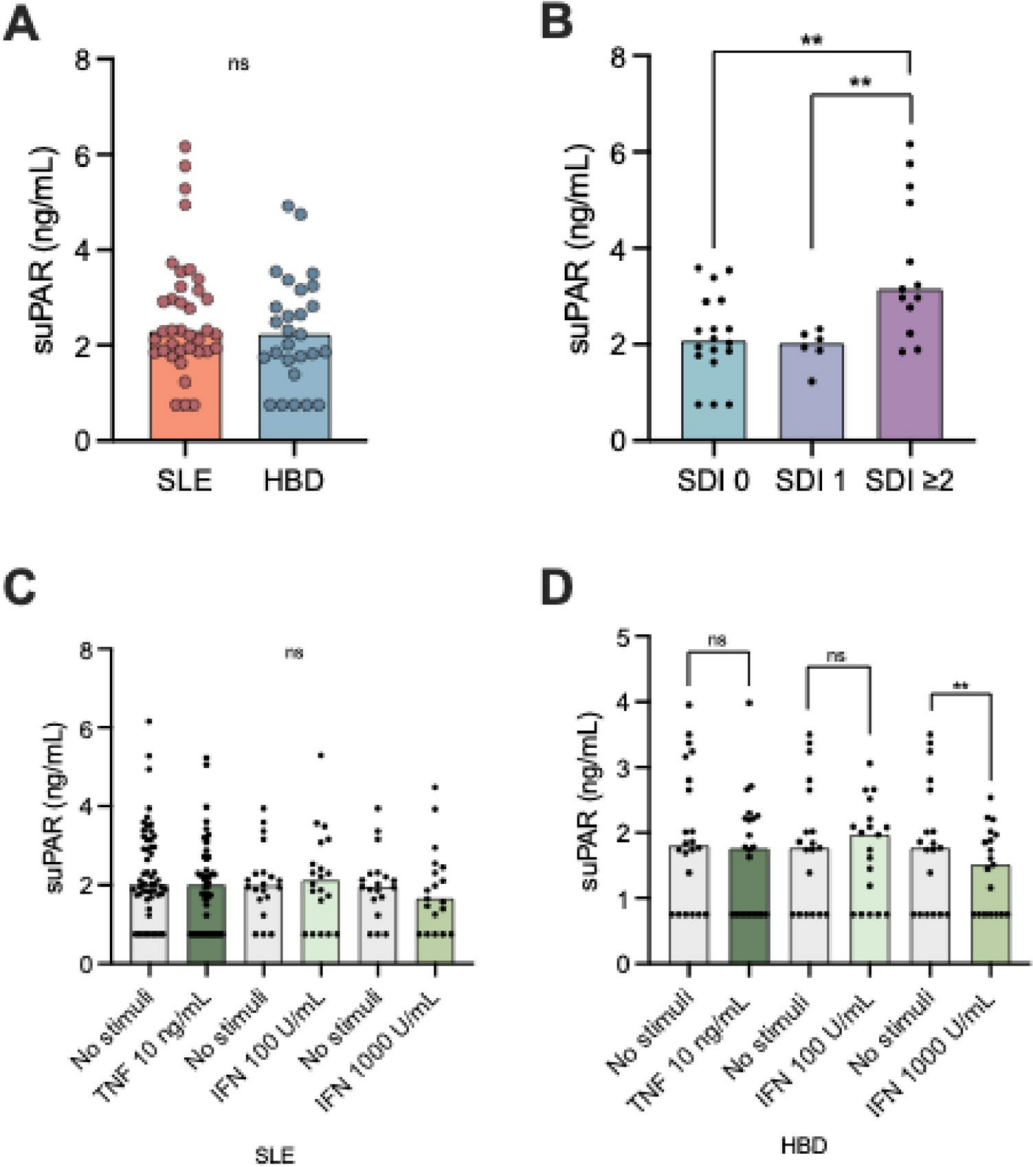
Soluble uPAR (suPAR) levels in patients with systemic lupus erythematosus (SLE) and healthy blood donors (HBD) (**a**), in patients with organ damage (**b**), after cytokine treatment in patients with SLE (TNF *n* = 54, IFN *n* = 20) and HBD (TNF *n* = 22, IFN *n* = 19) (**c** and **d**). TNF: tumor necrosis factor, IFN: Interferon, SLE: systemic lupus erythematosus, HBD: healthy blood donors. The graphs display median values as well as each individual. Significances from Kruskal–Wallis test are shown in (**b**) and from paired tests are shown in (**c** and **d**). **p < 0.001

Similarly to uPAR expression, no significant difference in unstimulated suPAR levels between patients and HBD was observed (Fig. 5a). No significant differences were found between SLE subjects meeting or not meeting the renal disorder (lupus nephritis) ACR criterion, or with daily intake of prednisolone dose equivalents (< 5 mg vs. ≥ 5 mg). However, patients with SDI ≥ 2 showed higher levels of suPAR in comparison with patients with less/no organ damage, and in comparison with HBD (Fig. 5b).

A significant correlation was found between age and suPAR level among the patients (rho = 0.458, *p* = 0.004) and between SLE disease duration and suPAR levels (rho = 0.556, *p* < 0.001). The impact of age, disease duration and SDI was further investigated in a linear regression analysis (stepwise approach) with 10log suPAR as the dependent variable. In this analysis, disease duration and age were removed from the model, leaving only SDI as the variable with a significant positive impact on suPAR levels. SDI was evaluated in the model both as a binary variable (SDI 0–1 vs. SDI ≥ 2; *p* < 0.001) and as continuous variable (*p* < 0.0001). No significant correlation was found between suPAR levels and age in the HBD group.

When examining the plasma suPAR levels before and after TNF treatment of whole blood, suPAR plasma levels were found to be unchanged for both patients and HBD (Fig. 5c and d). Treatment with IFN-α did not change the suPAR plasma levels in the patients with SLE (Fig. 5c). A significant decrease in suPAR was, however, only observed in HBD samples treated with the higher concentration of IFN-α (1000 U/mL), in comparison with the non-treated sample (Fig. 5d).

### Correlation between total uPAR cell surface expression and plasma suPAR levels

**Fig. 6:**
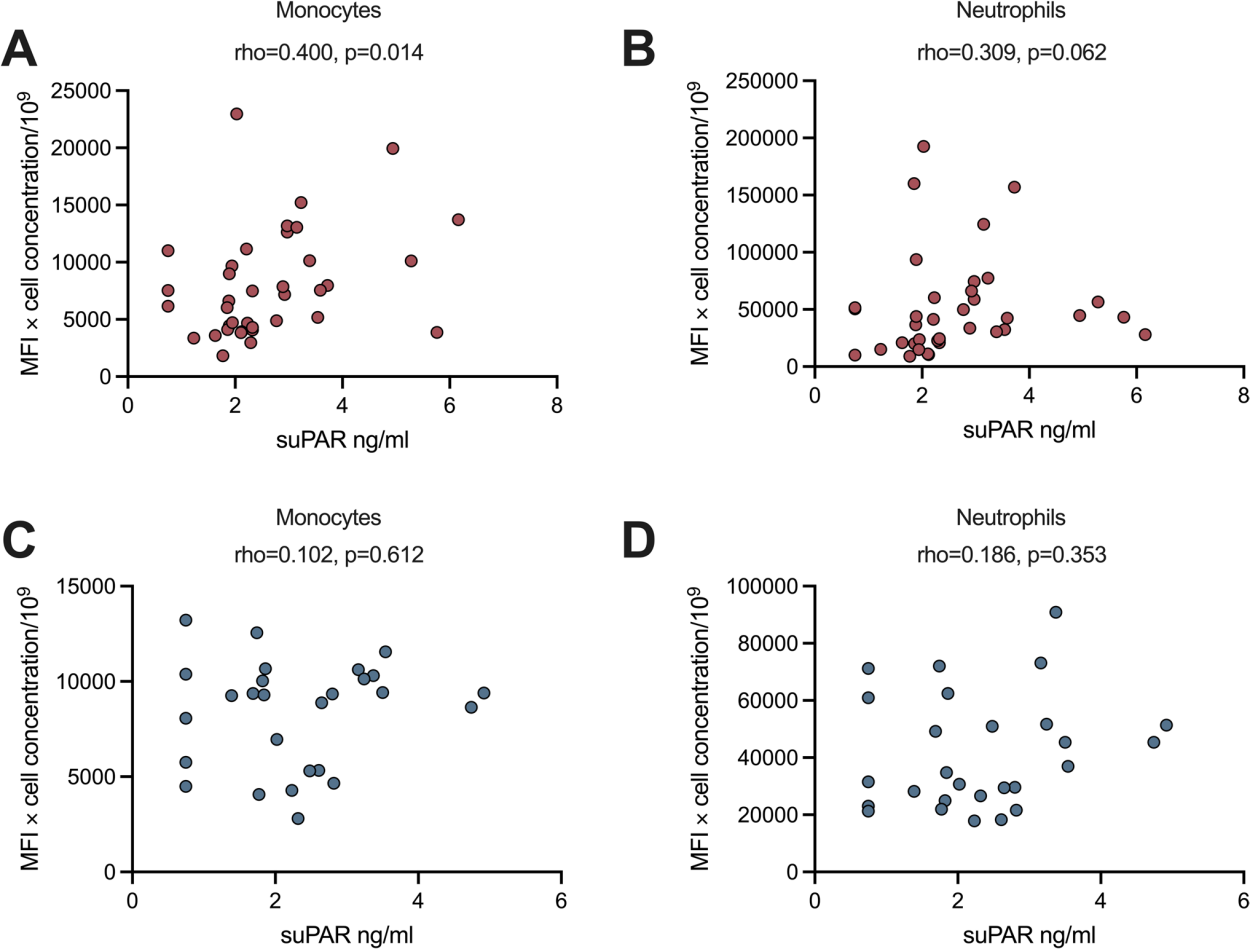
Correlation between total uPAR expression of monocytes or neutrophils with plasma suPAR levels in (**a** and **b**) patients with systemic lupus erythematosus (SLE) and (**c** and **d**) healthy blood donors

The total uPAR membrane expressions on neutrophils and monocytes (MFI × blood cell concentration), respectively, were correlated with suPAR levels for a better understanding of possible sources of circulating plasma suPAR. A significant positive correlation was observed between the unstimulated total expression of uPAR on monocytes in relation to baseline suPAR levels among the patients (rho = 0.400, *p* = 0.014), but significance was not reached for neutrophils (*p* = 0.062) (Fig. 6a and b). A separate correlation analysis for patients with organ damage (*n* = 19) revealed an even stronger correlation between total cell membrane uPAR expression on monocytes and plasma suPAR levels (rho = 0.565, *p* = 0.012). The same analysis on patients without damage (*n* = 18) did not reveal any significant correlation. Among HBD, there were no significant correlations between suPAR levels and total uPAR expression on any cell type (Fig. 6c and d). In cytokine-treated samples, no significant correlations were observed between suPAR and total uPAR expression (monocytes or neutrophils) among the HBD. However, among patients with SLE, a significant correlation was found between TNF-treated suPAR levels and total uPAR expression on monocytes (rho = 0.433, *p* = 0.013). No other correlations were found for treated samples among patients.

## Discussion

There is a gap of knowledge regarding the relationship between the cell-bound uPAR and the soluble form suPAR in inflammatory diseases. Herein, we investigated plasma suPAR and uPAR expression on untreated and treated leukocytes in HBD and patients with SLE. We observed no differences in the unstimulated cellular uPAR expression between patients and HBD. This was also true for plasma suPAR levels. Addition of TNF increased the expression of uPAR in both SLE and HBD on both monocytes and neutrophils, but the neutrophil upregulation was only seen in the activated neutrophil (CD11bhigh/CD62L^dim^) fraction. The addition of IFN-α, on the other hand, increased the cellular expression exclusively in SLE blood and did not induce a pronounced neutrophil activation. Although median plasma levels of suPAR were similar in HBD and subjects with SLE, the patients with organ damage accrual (SDI ≥ 2) exhibited significantly increased levels of suPAR. This was in line with our expectations based on previous observations [13], as well as suPAR’s ability to predict future organ damage [14]. Still, the monocyte/neutrophil plasma membrane expression of uPAR did not differ between patients depending on organ damage. This has not been shown before.

A significant correlation was found between total monocyte uPAR expression and the suPAR concentration in unstimulated samples from patients with SLE. This is interesting as IFN-α treatment resulted in upregulation of uPAR expression only in SLE samples, and most pronounced for monocytes. Even though IFN-α increased the monocyte and neutrophil expression of uPAR in SLE blood, there was no increase in suPAR plasma levels and no correlation between suPAR and cellular uPAR expression in IFN-α-treated samples. Most likely, the cells were already primed by type I IFNs (including IFN-α) as most patients with SLE display the so-called type I IFN gene signature characteristic of the pathogenesis [32]. Cells exposed to type I IFNs will increase their expression of type I IFN signaling components, which further augments their response to type I IFN stimulation [33]. This may also explain the differences in uPAR upregulation in response to IFN-α between patients and HBD. Conflicting with this assumption, however, is the moderate response to IFN-α among patients regarding neutrophil activation.

Neutrophils and monocytes are innate immune cells that both have been indicated in the pathology of SLE [34, 35]. Activated neutrophils have a crucial role in recruiting immune cells to inflamed tissues through the secretion of granule proteins. Among these proteins, two key ones are elastase and cathepsin G, which cleave uPAR into various forms of suPAR [36]. The gelatinase granules and primary granules contain uPAR, which are rapidly translocated to the plasma membrane upon activation and migration [37, 38]. Additionally, suPAR has been observed to be released rapidly following neutrophil activation with TNF [39]. Although neutrophils may be activated in different ways depending on stimuli, we were not able to show an increased suPAR release depending on the addition of TNF or IFN-α to the blood. In IFN-α-treated HBD blood, the suPAR concentration rather diminished, which is difficult to explain unless there is a rapid IFN-α-dependent degradation, cellular uptake or change in suPAR epitopes. The ELISA used in this study detects all forms of suPAR including full-length D_I_–D_III_ and the smaller D_I_ and D_II_D_III_ forms, and the results should thus not be confounded by the fragmentation of full-length suPAR.

IFN-α is central in the SLE pathogenesis, and recently, biological treatment targeting the IFN-α/β receptor (IFNAR) has been introduced to patients in clinical routine care [40]. IFNAR has a widespread tissue expression and is a prerequisite for type I IFN-dependent anti-viral effects. We were able to show increased uPAR on monocytes and neutrophils from IFN-α-treated patient blood but not in HBD. Effects of IFN-α on blood cell uPAR expression have, to our knowledge, not previously been shown and could also be of relevance in the context of other interferonopathies as well as in viral infections. Indeed, increased suPAR has shown to predict a worse outcome in COVID infections [23, 41].

TNF levels are increased in SLE, but the role of TNF in SLE remains controversial [42] with studies reporting associations with disease activity [43], but also induction of SLE or lupus-like disease as a result of exposure to anti-TNF agents [42]. Furthermore, an association between reduced anti-TNF autoantibody levels and lupus flares has been observed [44]. In the present study, TNF-stimulation resulted in upregulation of uPAR on monocytes and activated neutrophils but without a rise in suPAR levels. This corroborates findings in other studies showing TNF-induced uPAR expression on monocytes, without concomitant increase of suPAR [17, 45].

Neutrophils have been reported as the main source of shed suPAR in patients with sepsis, as an inverse correlation was demonstrated between uPAR surface expression and serum levels [24]. A positive correlation between plasma suPAR levels and uPAR cell surface expression on monocytes was found among the patients in this study, implicating different biological mechanisms behind raised suPAR in different diseases. Based on the results herein, we speculate that, in severe SLE (e.g., in subjects with acquired organ damage), there is a persistent upregulation of uPAR on monocytes, possibly under the influence of type I IFNs. At the same time, there is an increased shedding of uPAR into the circulation, giving a rise in suPAR. The circulating suPAR levels could also originate from other cell types, not studied herein (i.e., endothelial cells [46] and senescent cells [47]). Furthermore, an alternatively spliced uPAR mRNA, resulting in uPAR without a GPI-anchor, has been reported [48], indicating that suPAR may be produced directly without the need for cleavage from cell membranes. Plasma levels of suPAR are also under substantial genetic influence [49]. Altogether, this implies many possible routs for increased suPAR levels in SLE, other than increased shedding from monocytes.

Increased levels of suPAR in plasma, but foremost in urine, have been linked to lupus nephritis [1, 50], and upregulation of uPAR on podocytes has been associated with a variety of renal disorders with high levels of proteinuria (e.g., focal segmental glomerulosclerosis) [15]. Furthermore, bone marrow immature myeloid cells has been found as a key contributor of suPAR in chronic kidney disease in mice [51]. However, contrary to these findings, the current study reveals no upregulation of uPAR on neutrophils or monocytes in patients with renal disorders. Similarly, no significant difference in suPAR levels was found between patients with or without renal disorder. In the present study, 11 patients (30%) fulfilled the ACR criterion for renal disorder. The statistical power may thus be insufficient to detect significant differences.

One limitation of the present study is the lack of kinetic studies on uPAR expression, which implies a risk of missing important dynamics in the uPAR or suPAR response to TNF and IFN-α. Furthermore, we did not explore the exact mechanisms of action leading to uPAR upregulation and were not able to show that stimulated upregulation of uPAR is associated with increased suPAR levels. Strengths of the study include a well characterized patient material and an initial screening of uPAR expression on blood cells from patients with SLE before the in-depth experimental studies were performed. Furthermore, a clinically validated ELISA kit was used for the measurement of suPAR.

To conclude, as suPAR levels reflect and predict organ damage development in SLE, it is important to unravel its potential disease mediating effects as well as cellular sources. Results of this study suggest a different IFN-α-dependent regulation of uPAR expression in patients compared with HBD and show correlations between suPAR and monocyte expression of uPAR in SLE, especially in subjects with acquired organ damage. Together, this implies IFN-α as a regulator of uPAR expression and monocytes as a potential source of suPAR in SLE. Future studies should clarify the kinetic of cytokine-stimulated uPAR/suPAR and explore cytokine-mediated expression and shedding of uPAR in other tissues.

## Supplementary Information

Below is the link to the electronic supplementary material.

Supplementary file1 (DOCX 303 KB)

## Data Availability

No datasets were generated or analysed during the current study.
