# Supplementary material for: Interferon alpha-induced uPAR expression on monocytes as a potential source of increased soluble uPAR in patients with SLE at high risk of developing organ damage
Source: Clin Exp Med. 2025 Oct 21;25(1):317. doi: 10.1007/s10238-025-01897-4 (PMC12540527; doi:10.1007/s10238-025-01897-4)
Supplement: Supplementary file 1 — Supplementary file1 (DOCX 303 KB) [file 10238_2025_1897_MOESM1_ESM.docx]

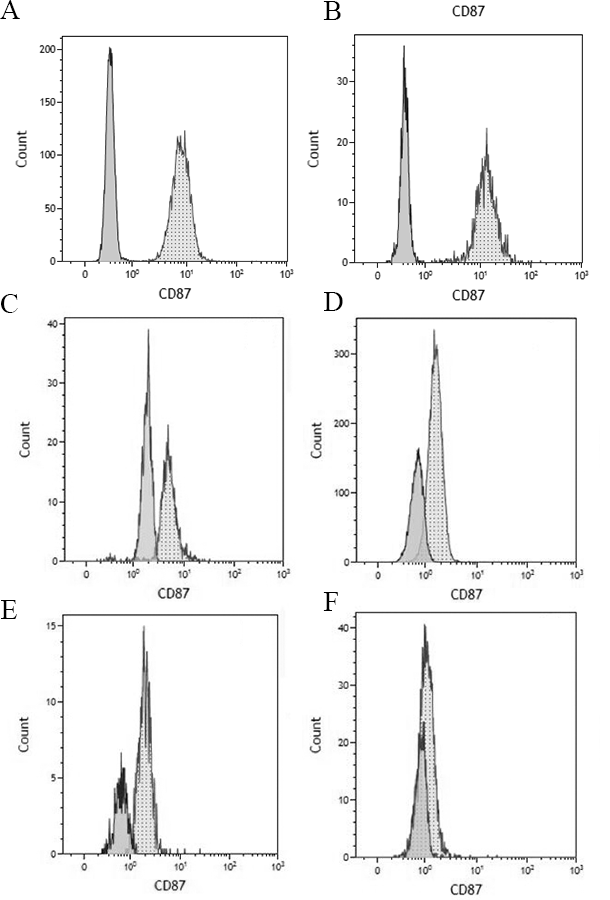


**Supplementary Figure 1. Cellular expression of CD87 by flow cytometry**. Representative graphs displaying the median fluorescence peak of BV421-conjugated anti-CD87 antibodies on neutrophils, monocytes, eosinophils, T-cells, B-cells, and NK-cells (Dotted A, B, C, D, E, F respectively) with fluorescence minus one (FMO) controls (Blank A, B, C, D, E, F respectively).


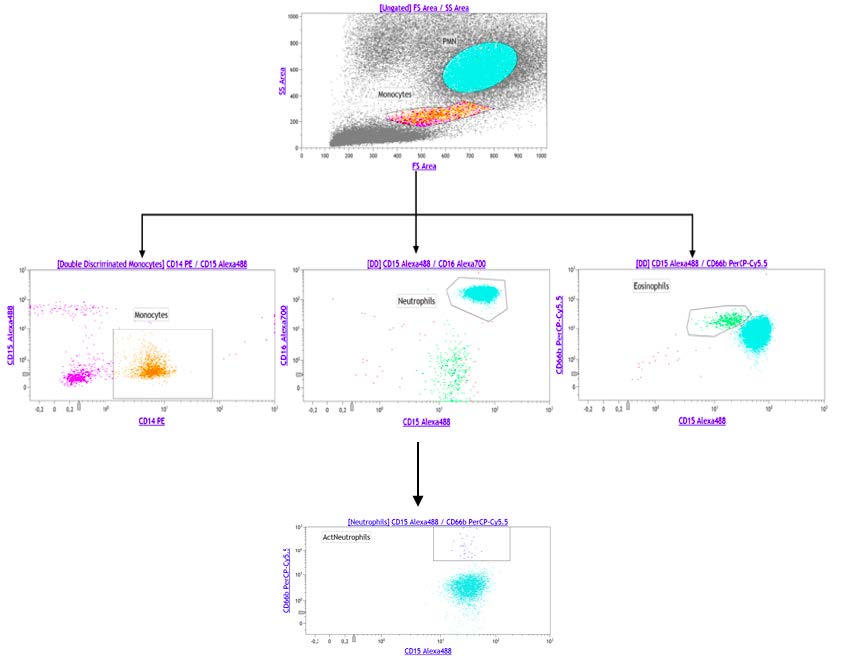


**Supplementary Figure 2. Gating strategy of neutrophils, monocytes and eosinophils.** Polymorphonuclear leukocytes (PMNs) and monocytes were located from total events using forward scatter (FS) area and side scatter (SS). Both PMNs and monocytes were gated through a double discrimination (DD) gate before defining neutrophils as CD15^+^/CD16^+^, monocytes as CD14^+^/CD15^-^, and eosinophils as CD15^+^ as a separate cloud of events (gated eosinophils were also seen as CD16^-^ distinct from neutrophils). Additionally, activated neutrophils were defined as CD66b^+^ neutrophils. Unstained cells, isotype controls and fluorescence-minus-one (FMO) controls were used in the set-up of the machine. Antibodies used: anti-CD87 (VIM5 clone, BV421 conjugated, IgG1; BD Biosciences, Franklin Lakes, NJ, US), anti-CD14 (M5E2 clone, PE conjugated, IgG2a; BD Biosciences), anti-CD15 (HI98 clone, Alexa488 conjugated, IgM; BioLegend, San Diego, CA, US), anti-CD16 (3G8 clone, Alexa700 conjugated, IgG1; BioLegend), anti-CD66b (G10F5 clone, PerCP-Cy 5.5 conjugated, IgM; BD Biosciences).


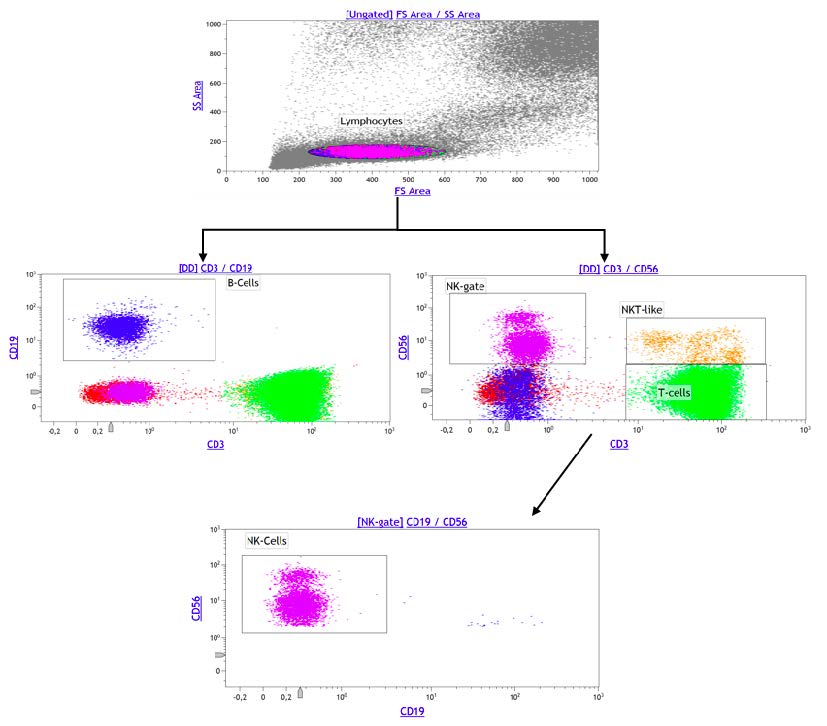


**Supplementary Figure 3. Gating strategy of T-cells, B-cells and NK-cells.** Lymphocytes were located from total events using forward scatter (FS) area and side scatter (SS). The lymphocytes were gated through a double discrimination (DD) gate before defining T-cells as CD3^+^/CD56^-^, B-cells as CD19^+^/CD3^-^, and NK-cells as CD56^+^/CD3^-^/CD19^-^. Unstained cells, isotype controls and fluorescence-minus-one (FMO) controls were used in the set-up of the machine. Antibodies used: anti-CD87 (VIM5 clone, BV421 conjugated, IgG1; BD Biosciences, Franklin Lakes, NJ, US), anti-CD3 (UCHT1 clone, Alexa 488 conjugated, IgG1; R&D Systems, Minneapolis, MN, USA), anti-CD19 (HIB19 clone, PE conjugated, IgG1; BD Biosciences), anti-CD56 (B159 clone, PerCP-Cy 5.5 conjugated, IgG1; BD Biosciences).
